# Therapeutic exercise following lumbar spine surgery: a narrative review

**DOI:** 10.1016/j.xnsj.2025.100620

**Published:** 2025-05-30

**Authors:** Ram Haddas, Andréas Remis, Yair Barzilay, Varun Puvanesarajah, Jake Keller, Brian M Clifford, Justin M. Lantz, John M. Mayer

**Affiliations:** a Department of Orthopedics, University of Rochester Medical Center, Rochester, NY, United States; b Department of Physical Therapy, Duke University, Durham, NC, United States; c Spine Unit, Department of Orthopedic Surgery, Shaare Zedek Medical Center, Jerusalem, Israel; d Integrated Spine Program, Department of Physiatry, Spine and Pain Management. Atrius Health—Part of Optum. Boston, Massachusetts, United States; e Division of Biokinesiology and Physical Therapy, University of Southern California, Los Angeles, CA, United States; f The Vert Mooney Research Foundation, San Diego, CA, United States

**Keywords:** Balance effort, Low back pain, Lumbar discectomy, Lumbar fusion, Risk of falls, Therapeutic exercise, Total disc replacement, Wearables

## Abstract

**Background:**

Therapeutic exercise is often part of the rehabilitation process following lumbar spine surgery. Despite this utilization, high-quality evidence and specific guidance regarding therapeutic exercise parameters and protocols following lumbar spine surgery remain sparse. The purpose of this manuscript is to provide a narrative review of the current evidence regarding exercise prescription, activity recommendations, and outcome measures following lumbar discectomy, fusion, and total disc replacement surgeries.

**Methods:**

A comprehensive narrative review was conducted regarding postoperative exercise for lumbar spine surgery, a summary of the current available literature was provided and interpreted, and future directions for research were identified.

**Results:**

The findings of narrative review generally support supervised therapeutic exercise as safe and beneficial following lumbar discectomy, fusion, and total disc arthroplasty surgeries. Initial exercise prescriptions should be individualized according to patient and surgical factors, including prior level of function, comorbidities, and tissue healing timelines, to support recovery goals. These findings can be useful for surgeons and rehabilitation practitioners to provide general guidance and to counsel patients on appropriate therapeutic exercises and activity recommendations following lumbar spine surgery, with a particular focus on the safety of exercise types and when to initiate exercise postoperatively. Physical activity monitoring (eg, wearables) can be used to complement postsurgical therapeutic exercise and enhance outcomes.

**Conclusions:**

This review provides the most current evidence regarding the safety and effectiveness of therapeutic exercise following lumbar spine surgery. Given the narrative format of this review, these findings are not intended to serve as clinical practice guidelines or provide specific recommendations about implementation. Thus, pragmatic implementation of exercise following lumbar spine surgery depends on the patient's distinct presentation, surgical factors, expertise and experience of the clinician, available resources, and patient preferences. Future prospective research trials and subsequent systematic reviews are needed to elucidate specific factors regarding the use of therapeutic exercise following lumbar spine surgery.

## Introduction

Therapeutic exercise is a prescribed physical activity modality that aims to improve symptoms and function [[1], [2], [3], [4], [5]] While it is common postsurgical practice to limit activity and exercise after lumbar spine surgery, if prescribed appropriately, postoperative therapeutic exercise can improve pain, disability, and function, and decrease opioid reliance through improving confidence, range of motion, strength, balance, and movement quality [3,[6], [7], [8], [9], [10]]. Despite this, the level of high-quality evidence regarding therapeutic exercise parameters and protocols following spine surgery remains sparse [3,[11], [12], [13], [14]], resulting in a wide variance in utilization [15,16]. The purpose of this manuscript is to provide a narrative review of the current evidence regarding exercise prescription, activity recommendations, and outcome measures following lumbar discectomy, fusion, and total disc replacement surgeries.

## Methods

*Information Sources / Selection Process:* This manuscript is a component of the North American Spine Society (NASS) Section's effort to develop a series of papers for a focus issue in the NASS Journal. Each NASS section was asked to produce a manuscript summarizing the current state of the art and future directions of the section's subspecialty area, in the format of a review article. In response to this call, the current review was prepared by certain members of the NASS Section on Interdisciplinary Spine, along with invited experts in the field. The authors of this manuscript consisted of an interprofessional team of spine care clinical specialists and researchers in the fields of biomechanics, rehabilitation sciences, physical therapy, orthopedic surgery, exercise science, and chiropractic care. Each author was asked to produce content for the review according to their expertise, relying heavily on peer-reviewed publications for available evidence.

*Design and Eligibility Criteria:* A comprehensive narrative review was conducted regarding postoperative exercise for lumbar spine surgery. We identified relevant peer-reviewed literature by using a comprehensive search strategy on PubMed and Google Scholar, which included the search terms “low back pain,” “rehabilitation,” “lumbar spine,” “exercise,” “activity restrictions,” “outcome measures,” and “spine surgery.” Two authors screened abstracts, and those that met the inclusion and exclusion criteria were included for the narrative review. If disagreement existed on the inclusion or exclusion of an available study, the first and last author made the decision regarding eligibility. Studies were included if they assessed nontraumatic, nonpathologic low back pain with various diagnoses (eg, disc herniation, degenerative disc disease, arthrosis, radiculopathy, stenosis, spondylolisthesis). Studies assessing low back pain related to trauma, tumor, infection, other pathologies, and spinal deformity were excluded. Surgeries included lumbar discectomy, fusion, and total disc replacement procedures. Exercises included various types delivered postoperatively, such as trunk-specific, functional training, and general physical activities. Following the search strategy and screening, all authors reviewed the resulting content and provided feedback until a consensus was reached. The first author and last author resolved differences, as needed. Results are organized into the following sections: General Considerations, Exercise Prescription, Components of a Postoperative Exercise Program, and Assessment Strategies.

## Results

### Postoperative exercise for lumbar spine surgery: general considerations

*Discectomy:* Therapeutic exercise is supported after discectomy to improve function, disability, pain, return to work duration, quality of life, and muscle strength, with functional benefit demonstrated at twelve-year follow-up compared to no treatment or massage [2,4,17]. Beginning therapeutic exercise immediately or within 2, 4, 6, and 8 weeks of discectomy appears beneficial without increasing adverse events or reoperation rates. Supervised programs provide greater benefits than nonsupervised exercise or advice [3,[17], [18], [19], [20], [21]]. Restrictions of bending, lifting, and twisting for 2 weeks versus 6 weeks did not change outcomes 1 year postdiscectomy [22]. In those not given postoperative restrictions, the average return to work duration was 1.2 weeks, and return timing did not affect complication rates [10]. A summary of the available evidence regarding the initiation of physical activity tasks following lumbar discectomy is provided in Table 1.

**Table 1:** Summary of available evidence regarding physical activity task timeframes following lumbar discectomy, fusion, and total disc replacement

	Timeframe from surgery to begin		
Activity	Discectomy	Lumbar fusion	Total disc arthroplasty
Ambulation	Immediately [210]	Immediately [[210], [211], [212]]	Immediately [61]
Initiate therapeutic exercise	Immediately [20]	3 weeks [25]	2 weeks [61]
Lumbar spine flexion, side bending, rotation	2 to 6 weeks [22]	Historically, twelve weeks [212]	Six weeks (+ no hyperextension) [30,61]
Swimming	Six weeks with healed wound [213]	Six weeks with healed wound [213]	Six weeks with healed wound [30]
Physical work involving lifting	Eight weeks [29,214]	Twelve weeks [214]	Six to twelve weeks [29,61]
High risk activities and competitive sports	3 months [214]	Six months [214]	Six months [30]

*Lumbar Fusion:* Therapeutic exercise following lumbar fusion can improve pain and disability at 6 months without increasing adverse events compared to usual care [[23], [24], [25]]. The benefit is furthered if supervision and multimodal interventions (eg, psychosocial components) are provided [6,8,23,26,27]. 29% of patients receive supervised exercise in the first year following single-level lumbar fusion [15]. Those who receive supervision have higher complexity (eg, fusion levels, operation duration, hospital stay), yet achieve similar outcomes as lower-complexity cases [28]. Onset of exercise at twelve weeks postoperatively may provide greater outcomes than at 6 weeks [8]. Certain exercises (ie, isometric trunk strengthening) initiated at 3 weeks provide greater functional outcomes than usual care at 3 months [25]. The median return to work duration following lumbar fusion is ten weeks, regardless of work intensity [29]. A summary of the available evidence regarding the initiation of physical activity tasks following lumbar fusion is provided in Table 1.

*Total Disc Arthroplasty:* Beginning therapeutic exercise 3 weeks following total disc arthroplasty improves outcomes without increasing adverse events, with supervision and increased frequency associated with greater improvements [7,30]. A summary of the available evidence regarding the initiation of physical activity tasks following lumbar total disc replacement is provided in Table 1.

### Postoperative exercise for lumbar spine surgery: exercise prescription

Our review found no clinical practice guidelines for therapeutic exercise prescription following lumbar surgery, which is consistent with other work [13,16,31]. Therefore, exercise prescription should be individualized based on patient and surgical factors, including patient goals, demographics, surgery date and type, and tissue-healing timelines [32,33]. Generally, early-onset exercise improves outcomes at 12 to 18 months postoperatively compared to standard onset [34]. Individualizing care is paramount to meeting the unique needs of each patient, as standardized group exercise programs do not provide additional benefits to usual care following lumbar surgery [35].

*Patient-related Factors:* Prior level of function and biopsychosocial factors are patient-related factors to consider when prescribing postoperative exercise [[36], [37], [38]]. Assessment of prior level of function, including comorbidities and recent activity levels, allows for consideration of tissue readiness for load [39]. Comorbidities increase complication rates [40]. Acute to chronic load ratios examine workload over the last week compared to the last month; higher acute to chronic load ratios may increase injury risk [41,42]. Low baseline physical activity is common in those with persistent spine pain and significantly reduces adherence to exercise prescriptions [43,44].

Biopsychosocial factors, which consider the multifactorial nature of low back pain, are a strong driver of persistent pain and affect participation in therapeutic exercise [[44], [45], [46], [47]]. For example, high fear avoidance towards physical activity is associated with worse outcomes 1 year following lumbar surgery [48]. It was demonstrated in a prospective cohort that early feelings of fear avoidance and depression in the early postoperative phase (<6 weeks) predicted pain intensity, pain interference, disability, and physical health at 6-month follow-up [49]. Cognitive behavioral therapy components appear to improve pain, function, and performance-based tests more than education alone postoperatively [50]. Screening tools such as OSPRO-YF may be utilized to support clinicians, as the identification of psychosocial factors through clinical assessment alone is limited [[51], [52], [53]].

*Surgery-related Factors:* Type, date, and approach of surgery, complications, and surgery-specific tissue-healing timelines are surgery-related factors to consider when prescribing postoperative exercise [54,55]. Specific muscle groups and incision healing may need consideration according to the surgical approach (anterior, lateral, posterior) [56]. Following lumbar discectomy, the annular defect requires time to scar together [57], with larger defects increasing re-herniation rates [40,58]. Some studies demonstrate no adverse events with restriction-free recovery following discectomy; however, patients may have naturally avoided certain movements [59]. Therefore, 6 weeks is often provided as a common timeframe for postdiscectomy activity restrictions [22]. In lumbar fusion, 50% of fusion occurs by postoperative week 5 and the remaining majority by week twelve [60]. This correlates to common postoperative bending, lifting, and twisting restrictions of twelve weeks. In lumbar total disc arthroplasty, the implant requires time to adhere to the end plates, explaining the recommendation of no lumbar hyperextension for 6 weeks [61].

### Components of a postoperative exercise program

Range of motion, muscle function, balance, and movement quality should be considered in postoperative exercise prescription as they may contribute to activity limitations and participation restrictions [62]. The type, intensity, and progression rate of impairment-based prescriptions will depend on patient and surgical factors as previously described; progression under clinician guidance is recommended, and strength and conditioning principles should be applied [63].

Range of Motion: The physical stress theory suggests that biological tissues predictably adapt to physical stress [64]. Similarly, mechanotherapy and mechanotransduction consider adaptive cellular responses to physical stimulation [[65], [66], [67]]. Bone responds to appropriate load with increased density, and ligaments respond to tensile load with increased tensile strength [68,69]. A small percentage of available lumbar motion is used for most activities of daily living, except for picking up an object from the ground [70]. Addressing range of motion limitations above and below the surgical region may decrease strain through the healing lumbar spine [[71], [72], [73]]. Special consideration may be made for hamstring motion, as a common surgical indication is neuropathic leg symptoms involving the sciatic nerve [74]. Surgery decompresses the involved nerve roots, but nerves may require time to recover before exposure to the strong tensile forces of traditional static stretching [75,76]. A recent systematic review provides moderate-level support for neural mobilization in those with a history of lumbar spine surgery [77].

Muscle Function: Arthrogenic trunk muscle inhibition is found in those with lumbar pain and injury, and trunk muscle function is moderately correlated with disability following lumbar surgery [78,79]. Trunk strength does not spontaneously normalize following lumbar surgery [78,80]. Those undergoing surgery demonstrate worse trunk strength than norms preoperatively and at 1- and 5-year postoperative follow-ups and poorer preoperative muscle quality prolongs postoperative strength recovery [78,[80], [81], [82]]. Endurance testing assesses symmetry across trunk musculature; expected norms are extension to flexion ratios of >1.00, extension to lateral flexion ratios of >0.75, and lateral flexion symmetry of >95% [83,84].

Trunk muscle function can be split into local and global approaches [83,85]. A local approach focuses on specific muscle activation (eg, multifidus, transverse abdominis), and a global approach focuses on activation as a collective unit [85,86]. When comparing approaches, meta-analyses demonstrate no difference in outcomes; similar benefits are also found following discectomy [83,87,88]. Research supports the abdominal draw-in as a safe entry point for trunk muscle activation following lumbar spine surgery [89]. Contraction intensity can be modulated, and appropriate performance induces minimal spinal movement [90]. Isometric performance can be progressed to various body positions with external perturbations to develop trunk endurance, such as bilateral shoulder flexion or modified Roman chair variants [84,89]. Uniplanar challenges are appropriate early for isolated muscle strengthening and triplanar later to match the co-contraction demands of life [91].

Lower extremity strength is impaired in those with low back pain, and improving lower extremity strength improves outcomes [92,93]. Exercises such as prone hip extension and side-lying hip abduction demonstrate lower stress through the spine than step-ups and sit-to-stands, suggesting utility in early rehab and for those with higher symptom severity [[94], [95], [96]]. Special considerations are placed toward muscles with reduced strength associated with a nerve root myotome. Myotomal deficit from nerve root compression is a common surgical indication and should be assessed postoperatively [97,98]. Myotomal deficits typically recover within 3 months of surgery but can take up to 1 year [99].

Balance: Those with low back pain have impaired balance, including decreased balance efficiency, gait speed, step length, gait consistency, and ability to recover from falling [88,[100], [101], [102], [103]]. Lumbar surgery or pain relief does not appear to fully restore balance compared to healthy controls, indicating appropriateness of postoperative balance training. Therapeutic balance exercises decrease fear of falling [[104], [105], [106], [107]]. Reactive balance is the last defense against a fall, and single-task challenges do not transfer to dual-task ability; these unique considerations should be trained accordingly [108,109].

**Fig. 1:**
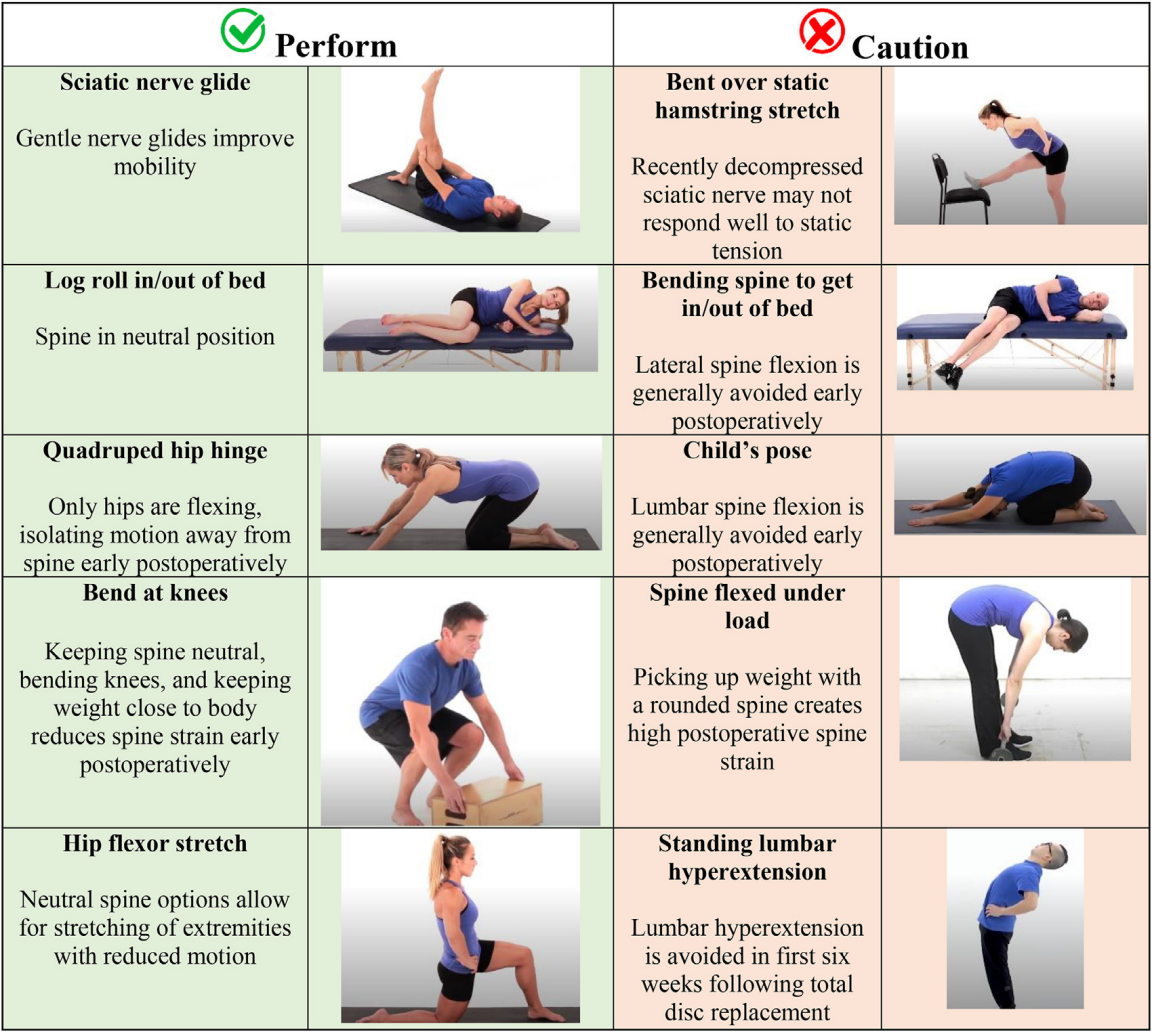
Examples of exercises to perform and be cautious of in early-recovery following spine surgery.

Movement Quality: Movement quality describes the movement patterns a patient demonstrates [110]. Examples include fluidity, symmetry, and movement selection during activities, such as lifting items from the ground and transferring to and from a bed. Small changes can improve postoperative strain; for example, holding objects closer to the body when picking them up from the ground reduces spine stress [111]. Impairments in range of motion, strength, and balance may contribute to reduced movement quality [112,113]. For example, decreased hip flexion and ankle dorsiflexion motion during a squat may require greater lumbar flexion, which may be undesirable in the early postoperative period due to increased spinal load [111]. Other times, aberrant movement may occur despite normal motion and strength [114,115]. Therapeutic exercises should replicate movements during anticipated daily and vocational tasks [29]. Motor control exercises prescribed at <45% of maximal voluntary isometric contraction with external feedback, such as a quadruped hip hinge balancing object at the lumbar region and constraint-induced symmetrical squatting, can be helpful interventions to improve movement quality (see Fig. 1) [116,117].

Other Forms of Exercise: In addition to the specific therapeutic exercise principles described above, other forms of exercise of interest to the spine care provider may include Pilates, Yoga, and Taichi. There is sparse research available about using these exercise modalities following lumbar surgery. Pilates and Tai Chi provide similar effectiveness to other types of exercises for improving low back pain and disability, whereas Yoga, compared to no exercise, does not provide clinically significant improvements in persistent low back pain [[118], [119], [120]]. Movements performed in Pilates, Yoga, and Tai Chi are variable. While movement variability is desired in the long term, systematic progression should be considered. Returning to these activities could be viewed similarly to returning to a sport, where simulated performance of anticipated demands should be successfully performed under clinic supervision before resumption [121]. A summary of the available evidence regarding exercise prescription following lumbar spine surgery is summarized in Table 2.

**Table 2:** Summary of available evidence regarding exercise prescription after lumbar spine surgery

Factor	Recommendations
Intervention approach	1. Use therapeutic exercise, general physical activity, and education rather than passive agents [[215], [216], [217], [218], [219]]. 2. Address thoughts, feelings, and behaviors that interfere with engagement in appropriate amounts of therapeutic exercise to improve pain, function, and physical performance tests [48,50].
Exercise delivery method	3. Supervised exercise should include a home exercise program [220]. 4. Prescribe supervised therapeutic exercise to improve likelihood that higher complexity cases (eg, comorbidities, complications) achieve similar outcomes as lower complexity cases [3,28].
Exercise delivery timing	5. Encourage early postoperative exercise to reduce opioid use, disability, and pain, especially for those with high-risk factors (eg, kinesiophobia) [6,18,19,25,30,34,221].
Exercise modes and approaches	6. Exercises should target strength, range of motion, and balance, and movement quality [[215], [216], [217], [218], [219],^222^,^223^]. 7. Low tech (eg, floor-based) and high-tech (eg, machines) approaches provide similar outcomes; low tech may improve independent performance [[215], [216], [217], [218], [219],^222^,^223^].
Low preoperative activity levels	8. Keep acute to chronic workload ratios less than 1.5 to reduce injury risk [41]. 9. Abbreviate initial exercise program to improve exercise adherence [44]. 10. Reduce progression speed as poorer preoperative muscle quality prolongs postoperative strength recovery [82].
Restoring spinal range of motion	11. A conservative resumption of motion would be at 6 weeks following discectomy and disc arthroplasty, and at twelve weeks following fusion, according to postoperative tissue healing timeframes [[54], [55], [56], [57], [58],^60^,^61^]. 12. Initiate and progress motion from uniplanar to multiplanar, small to large amplitude, and reduced to normal weight-bearing for appropriate graded exposure to required compressive and tensile forces of life [64]. 13. Picking up an object from the ground is the daily activity that requires the most amount of lumbar motion; clinicians should proactively counsel patients early postoperatively on appropriate strategies [70].
Restoring extremity range of motion	14. Initiate early in neutral-spine positions using one 30-second static stretch per day for each limited plane of motion to decrease strain and compensation through the lumbar spine [[71], [72], [73],^224^,^225^]. 15. Perform light sciatic nerve sliding mobilizations rather than static hamstring stretching in those recovering from radiculopathy to decrease nerve stain and increase longitudinal nerve excursion [75,76,226].
Restoring trunk strength	16. Objectively assess progress and progressively overload strength challenges to reduce disability, arthrogenic muscle inhibition, and improve trunk strength [63,[78], [79], [80], [81], [82], [83], [84]]. 17. After trunk active range of motion has been restored, progress from isometric to isotonic variants and from uniplanar to triplanar to move from isolated strength building to co-contractions that appropriately match life demands [83,87,88,91].
Restoring extremity strength	18. Begin early, progressing from isolated open kinetic chain movements to compound closed kinetic chain movements to improve outcomes [[93], [94], [95], [96]]. 19. Clinicians should assess myotomal deficits over time to improve ability to identify complications over the postoperative period [227].
Restoring balance	20. Begin with static balance challenges that induce mild perturbation for target duration of 30 seconds before advancing challenge [228]. 21. Progress by manipulating base of support, vision, surface compliance, proactive or reactive components, and dual-tasking [108,109].
Restoring movement quality	22. Assess movement quality (eg, during gait, bed transfers, lifting, work tasks) and counsel patient on strategies to decrease spinal load during early postoperative period [111].
Return to recreational exercise and sport	23. Clearance from medical team and simulated performance of anticipated demands should be successfully performed under clinic supervision prior to resumption [121].

General Physical Activity: Physical activity is strongly supported for improving total and quality years of life and decreasing chronic disease risk [122,123]. Thus, postoperative recovery goals should include counseling on healthy physical activity levels. Common metrics for measuring physical activity include steps per day and activity intensity, which are correlated to outcomes following spine surgery [124,125]. Wearable technologies can improve data tracking of these metrics [126]. Participation in standard exercise regimens did not increase general activity metrics between 3 and 6 months postoperatively [127]. In contrast, simply using a wearable to monitor physical activity improves physical activity metrics [[128], [129], [130], [131], [132]]. Importantly, these data often provide quantitative and not qualitative metrics. Qualitative movement (eg, gait symmetry) should not be compromised to reach a quantitative metric.

Steps per Day: Early mobilization following surgery is widely supported [133]. At 6 weeks following spine surgery, those who walk more than 3500 steps daily are 4 times more likely to achieve an excellent outcome at 1 year (ie, ODI ≤20%, back and leg pain ≤2/10) and twice as likely to avoid opioids [125]. Those who participated in a progressive walking program following lumbar discectomy beginning with ten minutes 4 times per week improved disability and quality of life compared to the control group [134]. In the long term, all-cause mortality is decreased by 50% in those who ambulate 8000 steps per day compared to 4000 [135].

Activity Intensity: Higher-intensity exercise programs appear to provide more benefit than lower-intensity programs following lumbar spine surgery [19]. Intensity is often tracked via heart rate, with 64%–76% of max heart rate considered moderate-intensity activity [136]. Moderate-intensity activity can also be estimated using a metric of 100 steps per minute; about 3000 steps per day at a cadence of 100 steps per minute would meet the recommended 30 minutes of daily moderate-intensity activity needed to decrease risk of all-cause mortality by 75% [[137], [138], [139]]. Those who perform ≥150 minutes of moderate-intensity activity per week have a reduced risk of increased postoperative pain by 2-fold and need for revision surgery by 5-fold [124]. A 30% improvement in persistent low back pain and function is anticipated if performed as recommended for 12 weeks [140].

Unfortunately, at 6-month and 2-year follow-ups after lumbar surgery, 0% and 24% of patients achieve ≥150 minutes of moderate-intensity activity per week, respectively [124,127]. Therefore, counseling on and tracking of activity intensity should be a component of postoperative care. A summary of the available evidence regarding activity recommendations and benefits following spine surgery is summarized in Table 3.

**Table 3:** A summary of available evidence regarding activity-recommendations and benefits following spine surgery

Activity-recommendations	Early-postoperative (by 6 weeks)	Late-postoperative (6–12 weeks)	Benefits if achieved
Steps per day	3500	8000	Four times more likely to achieve an excellent outcome [125] 50% reduction in all-cause mortality [135]
Activity-intensity	Progressive walking program beginning with 10-minutes 4 times per week.	≥150-minutes per week of moderate-intensity activity (measured via 64-76% max-heart rate or >100 steps-per-minute for 3000 steps)	Decreased risk of increased postoperative pain by 50% [124] Five times less likely to need revision surgery [124] 75% reduction in all-cause mortality [139] 30% improvement in persistent low back pain and function if performed for twelve weeks [140]

Note: activity recommendations are quantitative and not qualitative metrics; consult a clinician for guidance in safely achieving the recommendations.

**Fig. 2:**
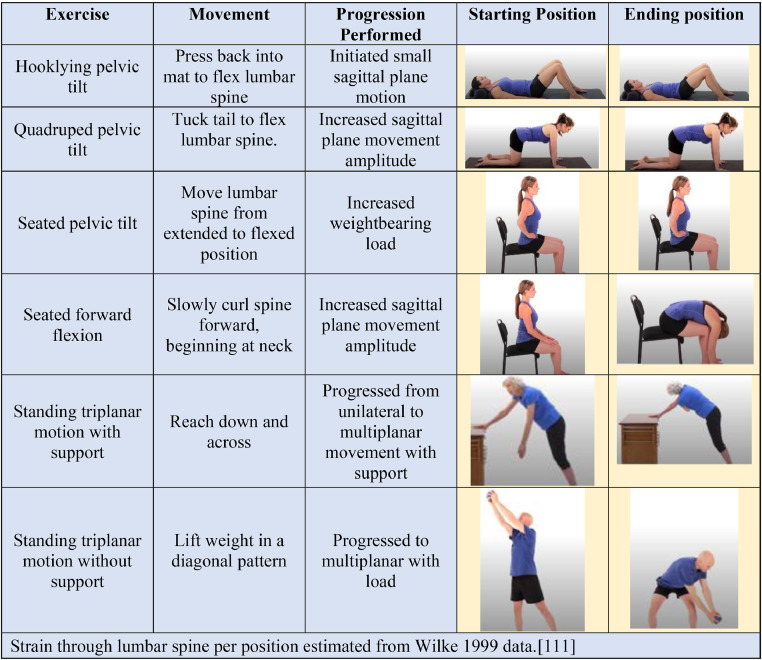
Example of graded-progression of lumbar spine motion.

Considerations regarding Activities to Perform and Avoid: Clinical decision-making about the safety of physical activities following spine surgery is crucial for postsurgical exercise prescription, as postoperative restrictions vary significantly [3,[11], [12], [13]].. All activities have inherent benefits and risks, with patient and surgical factors affecting the balance [141]. In the early postoperative period, activities may be modified for 6 weeks following discectomy and total disc replacement and for twelve weeks following fusion (Table 1) [127]. Generally, graded exposure to activities using incremental progression while respecting tissue healing timeframes is more appropriate than immediately returning to an activity at a given chronological milestone (Fig. 2) [32,56,60,142,143].

While caution in the early rehabilitation period is reasonable (Fig. 1), activity restrictions should be temporary as long-term adherence to restrictions may be unnecessarily restrictive. For example, research demonstrates that training in lifting techniques or lifting with a flexed lumbar spine does not change risk, recurrence, or persistence of low back pain [[144], [145], [146]]. Rather, injury may result more from exceeding tissue capacity secondary to inadequate work-to-recovery ratios or too abrupt a load increase [41,42,147]. Therefore, progressive exposure to anticipated life demands to develop appropriate capacity, including movement variability, is preferred. Future research using data from wearable devices may offer greater insight into associations between postoperative activities and adverse events.

### Assessment strategies for postoperative exercise and physical activity after lumbar spine surgery

Spine care providers often report success of postoperative exercise based on outcome measurements. Postoperative outcomes may include patient-reported, physical capacity, or physical performance measures [[148], [149], [150], [151], [152]]. Data analysis from these measures provides a robust assessment of a patient's disease severity, intervention effectiveness, and rehabilitation progress [148,152,153].

Patient-reported and Physical Capacity Outcome Measures: Patient-reported and physical capacity outcome measures quantify progress at a given point postoperatively [102,126,149,153,154]. Patient-reported measures include Numeric Pain Rating Scale, PROMIS, Patient-specific Functional Scale, Oswestry Disability Index, and Roland-Morris Disability Questionnaire (Table 4) [[155], [156], [157], [158], [159]]. Physical capacity measures include gait speed, Timed-up and Go, 6-minute Walk Test, 30-second Sit to Stand, trunk endurance tests, Functional Reach Test, Single-leg Balance, and Romberg’s test (Table 5) [84,[160], [161], [162], [163], [164], [165]].

**Table 4:** Patient-reported outcome measures

Patient-reported outcome measures	Type of assessment	Metrics
Visual Analog Scale (VAS)	Pain	MCID: 2 [229]
Numeric Pain Rating Scale	Pain	MCID: 2 [230]
PROMIS-Pain Interference	Pain	MCID: 8 [231]
PROMIS-Physical Function	Function	MCID: 8 [231]
Patient-specific Functional Scale	Function	MCID: 1.4 [232]
Oswestry Disability Index (ODI)	Disability	MCID: reduction of presurgical score by 30% (30% lower than presurgery score, not absolute reduction by 15 points) [233].
Roland-Morris Disability Questionnaire	Disability	MCID: reduction of presurgical score by 30% (30% lower than presurgery score, not absolute reduction by 15 points) [234].
Tampa Scale of Kinesiophobia (TSK)	Psychological	MCID: 6 [149,235]
Fear-Avoidance Beliefs Questionnaire (FABQ)	Psychological	MCID: 13 [149,236]

**Table 5:** Physical capacity outcome measures

Physical capacity outcome measures	Type of assessment	Metrics
Gait Speed	Mobility	Fall risk: <0.7 m/s [237] MCID: 0.1 m/s
Timed-up and Go	Mobility	Fall risk: >12 s [238] Severe impairment: >18.4 s [239] With manual dual-task: >14.5 s With cognitive dual-task: >15.0 s MCID: 2.0 s [240]
6-minute Walk Test	Endurance	MCID: 57.5 m [241] Normative age/sex data available [242]
5-times Sit to Stand	Lower-extremity strength and power	Fall risk: >12 s [238]
30-second Sit to Stand	Lower-extremity strength and muscle endurance	Normative age/sex data available [243,244]
Anterior Trunk Endurance Test	Anterior-trunk endurance	Normative Data Available Male: 144 s Female: 149 s [84]
Forearm Plank Test	Anterior-trunk endurance	Normative data available Male: 124 s Female: 83 s [245]
Sorensen Test	Posterior-trunk endurance	Normative Data Available Male: 146 s Female: 189 s [84] Positive back pain: 95 s Negative back pain: 133 s [246]
Lateral Trunk Endurance Test	Lateral-trunk endurance	Normative Data Available Male: 94-97 s Female: 72-77 s [84]
Functional Reach Test	Trunk control	Normative Data Available Community-dwelling older adults: 26.6 cm [247]
Single-leg Balance	Balance	Fall risk: <6.5 s [238]
Romberg’s Test	Balance	Narrow base of support, eyes closed, compliant surface: Fall risk: <20 s [248]
Gait Analysis	Mobility, Disability, Function, and Capacity	Gait Speed (Fall risk: <0.7 m/s), Cadance, Step length, step width, gait deviation index, join motion pattern, and gait compensation [88,153,154,249,250].
Cone of Economy - Balance Analysis	Balance, Disability, Function, and Capacity	Balance effort (Fall risk: > 60 cm), 3D Cone of Economy (CoE) dimension, balance compensation [100,102,103,149,153,195,249,251].
Dynamic Electromyography (EMG)	Disability, Function, and Capacity	Neuromuscular activity, muscle onset, muscle symmetry, co-contraction [101,154,[251], [252], [253]].
Force Plate / Pressure Mat	Disability, Function, and Capacity	Ground reaction force (Fall risk: > 1.1 bodyweight), joint moment, joint power, pressure distribution [103,[254], [255], [256]].
Computer Dynamic Posturography	Balance, Disability, Function, and Capacity	Fall risk: SOT <60; CoE dimension > 6 cm; Visual, vestibular, and somatosensory systems, center of pressure, CoE [103]
Wearables	Disability, Function, and Capacity	Levels of activity, heart rate, and sleep time [126,192]

While these measures are an excellent source of data, they are also limited to a snapshot in time and are often infeasible to perform on a longitudinal daily basis. Patient-reported measures are subject to bias and perceptual mismatch [126,154,166,167]. Within-clinic physical capacity outcome measures demonstrate capacity in a controlled environment but do not offer data about a patient’s day-to-day physical performance [168]. Compared to preoperative metrics, patient-reported and physical capacity measures improved 6 months postoperatively, but real-life physical performance data did not [169].

Physical Performance Testing and Wearable Technology: Wearable technology is an emerging technology sector that can provide valuable health information to patients and clinicians through continuous physical performance data collection in real-life environments. Wearables can provide activity and spine-specific metrics using accelerometer and gyroscope data to estimate posture and balance [126]. The literature describes the use of wearables to evaluate postoperative changes in physical function following spine surgery [[169], [170], [171], [172], [173], [174], [175], [176], [177], [178], [179], [180], [181], [182], [183], [184], [185], [186], [187]]. Wearable-derived measures of physical function change in predictable patterns in response to clinical events: worsen as disease burden becomes more severe, remain low immediately after surgical intervention, and slowly improve over the postoperative recovery period. These patterns largely repeat across studies, with several authors noting that objective physical performance metrics improve more slowly than subjective patient-reported outcome scores (PROMs) [169,173,[175], [176], [177], [178], [179], [180], [181], [182], [183], [184], [185], [186], [187]]. Several authors concluded that no significant correlation exists between improvements in PROMs and changes in wearable-derived physical activity measurements, suggesting distinct benefits to monitoring physical performance measures [177,179,181,[183], [184], [185]].

As digital technology becomes more prevalent in orthopedics, it may revolutionize postoperative spine care through real-world physical performance feedback, improved patient-provider communication, and reduced healthcare costs and provider burnout [126]. Digital platforms and mobile health therapeutic exercise apps are becoming more prevalent and have demonstrated effectiveness in managing low back pain [188]. A randomized control trial demonstrated that a mobile health app intervention provided greater improvements in disability compared to usual care at 24-month follow-up following lumbar spine surgery [189]. Therefore, mobile health apps could improve postoperative disability in areas with limited regional healthcare facilities [190,191]. Additionally, the structure that a mobile health app provides may improve self-efficacy and sustainability of therapeutic exercise compliance compared to traditional physical therapy [191].

Technological Advances: High technology includes digital platforms, 2D and 3D motion capture, force platforms, inertial measurement systems, and electromyography [100,154,192]. For more sophisticated analysis of postoperative status, clinicians may order a gait, balance, and function analysis at a local human motion analysis/gait lab (Table 5). Clinical gait analysis is a clinically valuable biomarker and method to comprehensively quantify gait abnormalities [193]. This process is completed using specialized technology, including computer-interfaced video cameras and electrodes on the skin surface [154,194,195]. The data collected from these tools can be processed to accurately assess kinematic and spatiotemporal components of gait [153]. Quantitative gait analysis has been previously established as an objective functional assessment tool for spinal pathology [154]. In a gait lab, clinicians could expect a detailed report on kinematic (walking speed, step length and time, range of motion, range of sway), neuromuscular (contraction onset, magnitude, coordination), kinetic (ground reaction force and pressure), and physiological factors (heart rate, VO_2_ capacity) [102,103,154,195].

When assessing fall risk postoperatively, accurate measurement of the spine patient’s cone of economy (CoE) is important. Balance effort and CoE dimensions are typically measured in a clinical- or laboratory-safe environment with the Romberg test [102,195]. Previous literature suggests that large sway (ie, larger CoE dimensions) increases the risk of falls [102,153,195]. However, this does not always represent an individual patient’s full capacity for balance and consequent fall risk. A new alternative test, Computer Dynamic Posturography (CDP), has recently been developed to further characterize patients with balance disorders in stable and experimentally unstable environments and determine fall risk [103]. The results of this test indicate the root cause of fall risk (eg, visual, vestibular, and somatosensory), which can be used postoperatively to develop a patient-specific management plan [103]. While not currently prevalent, advanced functional analyses provide increased insight into postoperative objective deficits, progress, and potential contributing factors to continued symptoms.

## Discussion

The findings of this narrative review generally support supervised therapeutic exercise as safe and beneficial following lumbar discectomy, fusion, and total disc arthroplasty surgeries. While the literature discussed in this review is somewhat in contrast to the common clinical practice of activity restrictions after lumbar spine surgery, these findings can be useful for the surgeon and rehabilitation practitioner to provide general guidance and to counsel patients on appropriate therapeutic exercises and activity recommendations following lumbar spine surgery, with a particular focus on the safety of exercise types and when to initiate exercise postoperatively. Given the narrative format of this review, these findings are not intended to serve as clinical practice guidelines or provide specific recommendations about implementation. Thus, pragmatic implementation of exercise following lumbar spine surgery depends on the patient's distinct presentation, surgical factors, expertise and experience of the clinician, available resources, and patient preferences.

The findings of this review suggest that initial exercise prescriptions should be individualized according to patient and surgical factors, including prior level of function, comorbidities, and tissue healing timelines. In the early postoperative period, activities may be modified for 6 weeks following discectomy and total disc replacement and twelve weeks following fusion [196]. Exposure to activities using graded progression is more appropriate than immediately returning to an activity at a given chronological milestone [[196], [197], [198]]. Progressive exposure to anticipated life demands, including movement variability, is recommended. Overdoing exercise and activity can be counterproductive, further illustrating the importance of exercise program supervision by a spine care professional [198]. Assessment of progress using patient-reported, physical-capacity, and performance-based outcome measures allows for appropriate progress monitoring, including real-world activity. Return to activity or sport should be preceded by medical clearance, restoration of physical impairments, and successful supervised performance of anticipated demands [121]. The increased use of wearables and advanced laboratory movement analyses may allow for greater clarity about how postoperative exercise and activity affect outcomes.

Success rates with postoperative therapeutic exercise may be explained by several reasons. Lumbar surgery effectively decompresses nerves and, if needed, stabilizes the spine [199,200]. However, lumbar surgery may not address physical impairments of spine and extremity range of motion, muscle function, balance, movement quality, or psychosocial factors (eg, distress, self-efficacy, pain coping) [78,80,104,201,202]. Exercise reduces kinesiophobia, which 50% of patients experience following discectomy at 10 to 34-month follow-up [203,204]. Supervised exercise provides an opportunity to address thoughts, feelings, and behaviors that interfere with engagement in appropriate amounts of therapeutic exercise [48].

The nature of exercise requires a patient to actively participate in their health. Education alone does not appear to change low back pain risk, whereas exercise significantly decreases risk of future back pain and work absenteeism, and moderate strength evidence supports exercise as more beneficial than no care or passive treatment in managing persistent low back pain [[205], [206], [207]]. An individualized exercise program that improves physical impairments and psychosocial factors with counseling on appropriate progression back to desired activities may explain the postoperative benefits of exercise following discectomy, fusion, and total disc arthroplasty [2,4,17,[23], [24], [25],^30^].

The current body of evidence regarding therapeutic exercise following lumbar surgery has numerous gaps that need further research before wide-scale recommendations can be made. For example, the timing of exercise initiation postsurgically and other exercise prescription factors (eg, frequency, intensity, time, type) must be examined for various patient types and surgical procedures. Future research using data from wearable devices may offer greater insight into associations between postoperative activities and adverse events. Multimodal rehabilitation improves outcomes, yet current studies have not prescribed specific interventions for individualized psychosocial profiles [6,8,23,27,29]. Thus, future research should examine if tailoring interventions to specific psychological phenotypes improves outcomes [208,209]. Additionally, higher-level approaches for evidence synthesis (eg systematic review, health technology assessment) are needed to inform clinical practice.

## Conclusion

The findings of this narrative review generally support supervised therapeutic exercise as safe and beneficial following lumbar discectomy, fusion, and total disc arthroplasty surgeries. Initial exercise prescriptions should be individualized according to patient and surgical factors, including prior level of function, comorbidities, and tissue healing timelines. These findings can be useful for the surgeon and rehabilitation practitioner to provide general guidance and to counsel patients on appropriate therapeutic exercises and activity recommendations following lumbar spine surgery, with a particular focus on the safety of exercise types and when to initiate exercise postoperatively. Given the narrative format of this review, these findings are not intended to serve as clinical practice guidelines or provide specific recommendations about implementation. Thus, pragmatic implementation of exercise following lumbar spine surgery depends on the patient's distinct presentation, surgical factors, expertise and experience of the clinician, available resources, and patient preferences. Future prospective research trials and subsequent systematic reviews are needed to elucidate specific factors regarding the use of therapeutic exercise following lumbar spine surgery.

## Declarations of Competing Interests

The authors declare that they have no known competing financial interests or personal relationships that could have appeared to influence the work reported in this paper.
